# Observation of time-reversal symmetry breaking in the band structure of altermagnetic RuO_2_

**DOI:** 10.1126/sciadv.adj4883

**Published:** 2024-01-31

**Authors:** Olena Fedchenko, Jan Minár, Akashdeep Akashdeep, Sunil Wilfred D’Souza, Dmitry Vasilyev, Olena Tkach, Lukas Odenbreit, Quynh Nguyen, Dmytro Kutnyakhov, Nils Wind, Lukas Wenthaus, Markus Scholz, Kai Rossnagel, Moritz Hoesch, Martin Aeschlimann, Benjamin Stadtmüller, Mathias Kläui, Gerd Schönhense, Tomas Jungwirth, Anna Birk Hellenes, Gerhard Jakob, Libor Šmejkal, Jairo Sinova, Hans-Joachim Elmers

**Affiliations:** ^1^ Institut für Physik, Johannes Gutenberg-Universität Mainz, Staudingerweg 7, D-55128 Mainz, Germany.; ^2^ University of West Bohemia, New Technologies Research Center, Plzen 30100, Czech Republic.; ^3^ Sumy State University, Rymski-Korsakov 2, 40007 Sumy, Ukraine.; ^4^ Linac Coherent Light Source, SLAC National Accelerator Laboratory, Menlo Park, CA 94025, USA.; ^5^ Deutsches Elektronen-Synchrotron DESY, 22607 Hamburg, Germany.; ^6^ Ruprecht Haensel Laboratory, Deutsches Elektronen-Synchrotron DESY, 22607 Hamburg, Germany.; ^7^ Institut für Experimentalphysik, Universität Hamburg, 22761 Hamburg, Germany.; ^8^ Institut für Experimentelle und Angewandte Physik, Christian-Albrechts-Universität zu Kiel, 24098 Kiel, Germany.; ^9^ Institut für Experimentelle und Angewandte Physik, Christian-Albrechts-Universität zu Kiel, 24098 Kiel, Germany.; ^10^ Universität Kaiserslautern, Department of Physics, 67663 Kaiserslautern, Germany.; ^11^ Institute of Physics Academy of Sciences of the Czech Republic, Cukrovarnick’a 10, Praha 6, Czech Republic.; ^12^ School of Physics and Astronomy, University of Nottingham, NG7 2RD Nottingham, UK.

## Abstract

Altermagnets are an emerging elementary class of collinear magnets. Unlike ferromagnets, their distinct crystal symmetries inhibit magnetization while, unlike antiferromagnets, they promote strong spin polarization in the band structure. The corresponding unconventional mechanism of time-reversal symmetry breaking without magnetization in the electronic spectra has been regarded as a primary signature of altermagnetism but has not been experimentally visualized to date. We directly observe strong time-reversal symmetry breaking in the band structure of altermagnetic RuO_2_ by detecting magnetic circular dichroism in angle-resolved photoemission spectra. Our experimental results, supported by ab initio calculations, establish the microscopic electronic structure basis for a family of interesting phenomena and functionalities in fields ranging from topological matter to spintronics, which are based on the unconventional time-reversal symmetry breaking in altermagnets.

## Teaser

Time-of-flight momentum microscopy reveals unconventional time-reversal symmetry breaking in altermagnets.

## INTRODUCTION

Conventionally, two elementary classes of crystals with collinear magnetic order have been considered—ferromagnetic and antiferromagnetic. The ferromagnetic exchange interaction generates strong magnetization and spin polarization in electronic bands that break time-reversal (T) symmetry. Nondissipative Hall currents, including their topological quantum variants (*1*–*3*), as well as spin-polarized currents, vital in modern ferromagnetic information technologies (*4*–*7*), are all based on the strong Tsymmetry breaking in the electronic structure. However, ferromagnetic and topological insulating phases are poorly compatible, and the inherent magnetization of ferromagnets limits the capacity and speed of ferromagnetic spintronic devices. In the second conventional class, the antiferromagnetic exchange generates compensated collinear order with no magnetization. The resulting absence in antiferromagnets of strong Tsymmetry–breaking linear responses akin to ferromagnets has forced the antiferromagnetic spintronic research to exploit comparatively weak phenomena relying on relativistic spin-orbit coupling (*8*, *9*). The weak responses represent a roadblock. On the other hand, the zero magnetization is well compatible with materials ranging from superconductors to insulators, and it enables breakthroughs toward information technologies with ultrahigh capacity and speed (*10*–*12*).

**Fig. 1.**
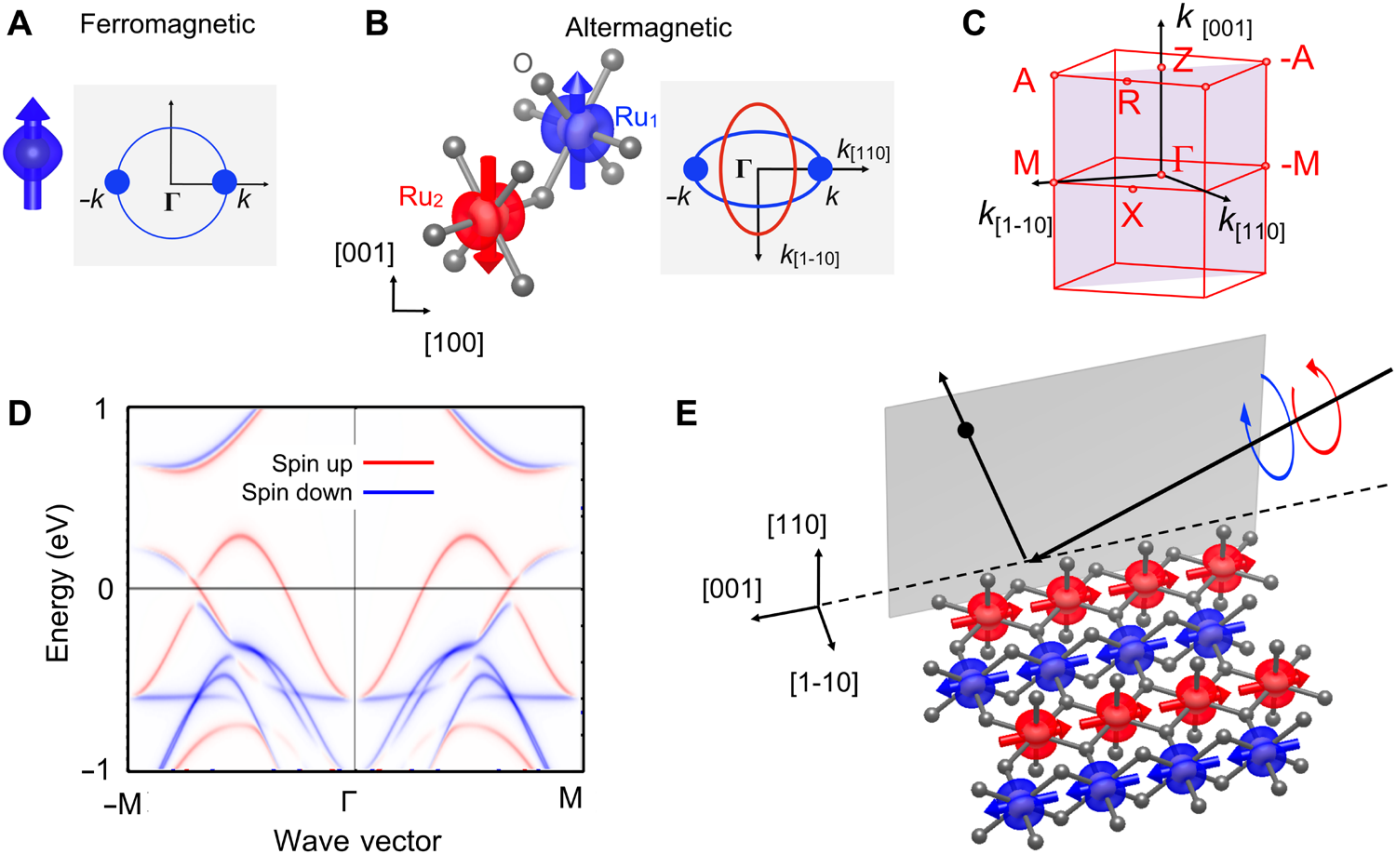
The spin-symmetry protected compensated antiparallel magnetic order in altermagnets generates an unconventional alternating spin polarization in momentum space. Comparison of (**A**) ferromagnetism, whose magnetization (left) generates conventional spin polarization and Tsymmetry breaking in the energy isosurfaces (right), and (**B**) altermagnetism, whose symmetry-protected compensated antiparallel magnetic order on a crystal (left) generates an unconventional alternating spin polarization and Tsymmetry breaking in the energy isosurfaces without magnetization (right) (*13*, *14*). Color-coding of bands in the momentum space reflects the spin orientation as depicted by arrows in the real space. Tsymmetry in the band structure is broken since time-reversed (opposite) momenta on the energy isosurfaces have non–time-reversed (same) spin orientations. In (B), the real-space model corresponds to RuO_2_, with gray spheres representing O atoms and color surfaces representing magnetization densities on Ru atoms. (**C**) Brillouin zone indicating the high symmetry points relevant for the spectra shown in the data. (**D**) Ab initio calculation of the Bloch spectral function of RuO_2_, showing a strongly broken Tsymmetry. (**E**) Sketch of the RuO_2_ magnetic crystal structure with the surface oriented along the [110] direction and the experimental setup of scattered photoelectrons from circularly polarized light.

The above examples illustrate why discoveries of magnetic quantum matter with unconventional characteristics and functionalities remain central to the frontier research in condensed matter physics and to the development of ultrascalable low-power technologies. Recently, a nonrelativistic spin-symmetry classification and description, focusing within the hierarchy of interactions on the strong Coulomb (exchange) interaction, has divided all collinear magnets into three mutually exclusive spin-group classes: (i) conventional ferromagnetic with strong (exchange) Treversal symmetry breaking in the electronic band structure and net magnetization, (ii) conventional antiferromagnetic with, at least in the nonrelativistic limit, Treversal symmetric electronic band structure and zero net magnetization, and (iii) a third class dubbed altermagnetic with strong (exchange) Treversal symmetry breaking in the electronic band structure and with, at least in the nonrelativistic limit, zero net magnetization (*13*, *14*). We note that the strong altermagnetic Tsymmetry breaking in the electronic structure is distinct from the previously studied relativistic spin-orbit coupling mechanism in conventional antiferromagnetic crystals with a symmetry combining Tand crystal inversion transformations (*15*), from weak ferromagnetism (*16*, *17*), or from noncollinear magnetism (*3*). Figure 1 illustrates the spin-symmetry protected compensated antiparallel magnetic order in altermagnets that generates an unconventional alternating spin polarization and the Tsymmetry breaking in the band structure without magnetization (*13*, *14*, *18*).

Altermagnets have been thus predicted to combine merits of ferromagnets and antiferromagnets, which were regarded as principally incompatible, and to have merits unparalleled in either of the two conventional magnetic classes (*13*, *14*). While unconventional anomalous Hall and spin-polarized currents have been predicted (*3*, *13*, *14*, *18*–*29*) and recently observed in experiment (*23*, *30*–*33*), so far there has not been a direct measurement of the underlying Tsymmetry breaking in the altermagnetic band structure.

A suitable microscopic tool is based on magnetic circular dichroism (MCD), whose optical version is the ac counterpart of the dc anomalous Hall effect. The presence of MCD in altermagnets over the full spectral range up to x-rays has been confirmed by ab initio calculations (*19*, *34*). Specifically, MCD in the angle-resolved photoemission spectroscopy allows for the microscopic visualization of the T symmetry breaking in the electronic structure in momentum space. In the past, this technique has been successfully used in the investigation of ferromagnetic materials (*35*–*43*).

Our experimental study focuses on epitaxial RuO_2_, a workhorse material of the altermagnetic class (*13*, *14*). The rutile crystal structure of metallic RuO_2_ has been shown to have a collinear compensated magnetic order (*44*, *45*) and predicted to be an altermagnet (*13*, *46*) with a strong (order eV) T symmetry breaking spin-splitting at certain momentum values, as shown in Fig. 1 (B and D). This rutile crystal family is predicted to show topological properties as well, with prior spin-integrated angle-resolved photoemission studies reporting two Dirac nodal lines and pronounced topological surface states (*47*). The predictions of strong anomalous Hall and spin currents in combination with vanishing magnetization (*18*, *26*) have also been experimentally verified (*30*–*33*) in this altermagnet.

Here, we present direct evidence for a strong T symmetry breaking in the band structure of epitaxial altermagnetic RuO_2_ films by detecting MCD in the angular distribution of photoelectrons, for both soft x-ray and ultraviolet photon excitation. We compare the experimental results to the corresponding calculations based on density functional theory. The measurements are done at the [001] orientation of the Ru moments, which by symmetry forbid the Anomalous Hall effect (AHE) and show no detectable remnant net magnetization, emphasizing the exchange origin of the altermagnetic lifting of the Kramer’s degeneracy observed.

## RESULTS

Using soft x-ray excitation, we can measure the intensity distribution of the direct transitions in four-dimensional energy-momentum space *I*(*E_B_*, *k_x_*, *k_y_*, *k_z_*), which is the spectral density function modulated by matrix elements accounting for the photoexcitation probability for a given initial *k_i_* and final state *k_f_*. As described in detail in the Supplementary Materials, for a given photon energy *h*ν and binding energy *E_B_* = *E* − *E_F_*, the final photoelectron states are located on a spherical shell with radius (for units Å^−1^ and eV)

$$k_{f}=0.512\sqrt{h\nu -E_{B}+V_{0}^{*}}$$ (1)

Here, the inner potential $V_{0}^{*}\approx 10$ eV is referenced to the Fermi energy, and the transferred photon momentum leads to a rigid shift of the free-electron final state sphere by the vector with absolute value *k_hν_* = 2π*ν*/*c* along the photon beam (*48*). The kinetic energy of the emitted photoelectrons is recorded by their time of flight, and the Fermi edge serves as reference for *E_B_* = 0. The photon energy range used in these experiments is 560 to 660 eV (see the Supplementary Materials for details).

In our measurements, *I*(*E_B_*, *k_x_*, *k_y_*, *k_z_*) is the intensity averaged between the two light polarizations. The intensity asymmetry, which contains the dichroism information, is calculated pixel by pixel as *A* = (*I*_+_ − *I*_−_)/(*I*_+_ + *I*_−_), with *I*_+_ and *I*_−_ denoting the intensity measured at circular right and left polarization. This intensity asymmetry contains a well-known dichroism component related to the measurement geometry and a component connected to the magnetic ordering. The geometry-related component is the so-called circular dichroism in the angular distribution (CDAD) (*49*, *50*), which is included in the asymmetric component of *A*(*k_x_*, *k_y_*) with respect to the line (*k_x_*, *k_y_* = 0) coinciding with the Γ − *Z* direction. CDAD is observed for a dissymmetric (handed) spatial arrangement of the quantization axis of initial state orbital momenta (*n*), the photon impact direction (*k_hν_*), and the photoelectron momentum (*k_e_*). Thus, CDAD from nonmagnetic targets requires a handedness in the experimental geometry. It is therefore strictly antisymmetric with respect to the plane of photon incidence spanned by *k_hν_* and the surface normal ([110] direction). To isolate the CDAD in the experimental data, we calculate the corresponding asymmetry as *A*_CDAD_(*k_x_*, *k_y_*) = [*A*(*k_x_*, *k_y_*) − *A*(*k_x_*, − *k_y_*)]/2.

In contrast to nonmagnetic systems, magnetic systems can provide an additional asymmetry mechanism if the light polarization vector is parallel or antiparallel to the spin axis, which gives rise to MCD (*35*). We can eliminate the contribution from the *A*_CDAD_ in the experimental asymmetry data and hence extract the MCD contribution in the remaining asymmetry by calculating *A*_MCD_ = [*A*(*k_x_*, *k_y_*) + *A*(*k_x_*, − *k_y_*)]/2.

**Fig. 2.**
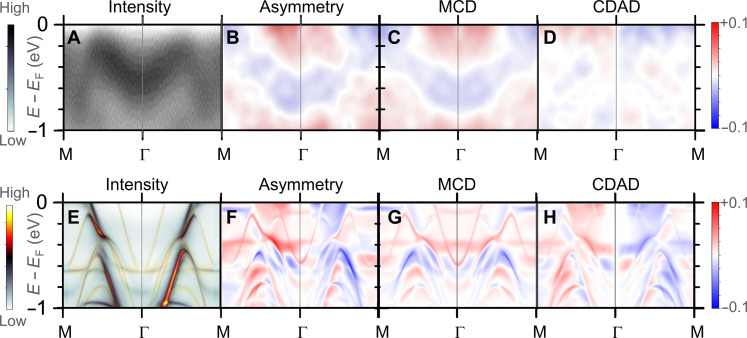
Experimental photoemission intensities and asymmetries are compared to calculated spectral densities. (**A**) Measured intensity map *I*(*E_B_*,0, *k_y_*) revealing the energy dispersion of bands along the line M-Γ-M (see Fig. 1C) measured at 70 K (see left color scale bar). (**B**) Measured intensity asymmetry *A*(*E_B_*,0, *k_y_*), (**C**) MCD *A*_MCD_(*E_B_*,0, *k_y_*), and (**D**) CDAD *A*_CDAD_(*E_B_*,0, *k_y_*) extracted from the asymmetry (see right color scale bar). (**E**) Calculated spectral density average (the thin yellow lines indicate the bands depicted in Fig. 1D) and (**F**) its asymmetry for circular right and left polarization of the incident light within the experimental geometry. The theoretical (**G**) MCD and (**H**) CDAD are extracted from the spectral density asymmetry as with the experimental MCD and CDAD. The photon energy used here is 660 eV. The maps are averaged over *k_x_* = ±0.1 Å^−1^.

We next present the key results of our studies in Fig. 1. We show the measured intensity *I*(*E_B_*,0, *k_y_*) along the M-Γ-M line (see Fig. 1C), the intensity asymmetry *A*(*E_B_*,0, *k_y_*), the MCD *A*_MCD_(*E_B_*,0, *k_y_*), and the CDAD *A*_CDAD_(*E_B_*,0, *k_y_*) in Fig. 2 (A to D), and their corresponding ab initio–based calculations for the specific experimental geometry in Fig. 2 (E to H). The theoretical density functional theory + U calculations are based on the one-step formulation of the photoemission process, using the Korringa-Kohn-Rostoker ab initio approach that represents the electronic structure of a system directly and efficiently in terms of its single-particle Green’s function (*51*, *52*). The parameters used in the calculations correspond to those in (*53*). We also note that the MCD spectra are not a direct map of the ground state polarization, as shown in (*54*), due to the final state effects. The calculations take into account the free electron–like final state at the corresponding *k_z_* (e.g., photon energy of 680 eV) and the matrix element of the induced transition.

As it is directly seen in Fig. 2 (C and G), the experimentally measured and theoretically calculated MCD spectra show a very strong T symmetry breaking whose magnitude is consistent with the exchange-dominated mechanism as predicted by the theory of altermagnetism (*13*). It is also important to contrast the MCD and CDAD data, which shows a dominance of the MCD contribution to the intensity asymmetry, confirming its direct observation beyond any experimental artifact that may have originated from the CDAD signal.

**Fig. 3.**
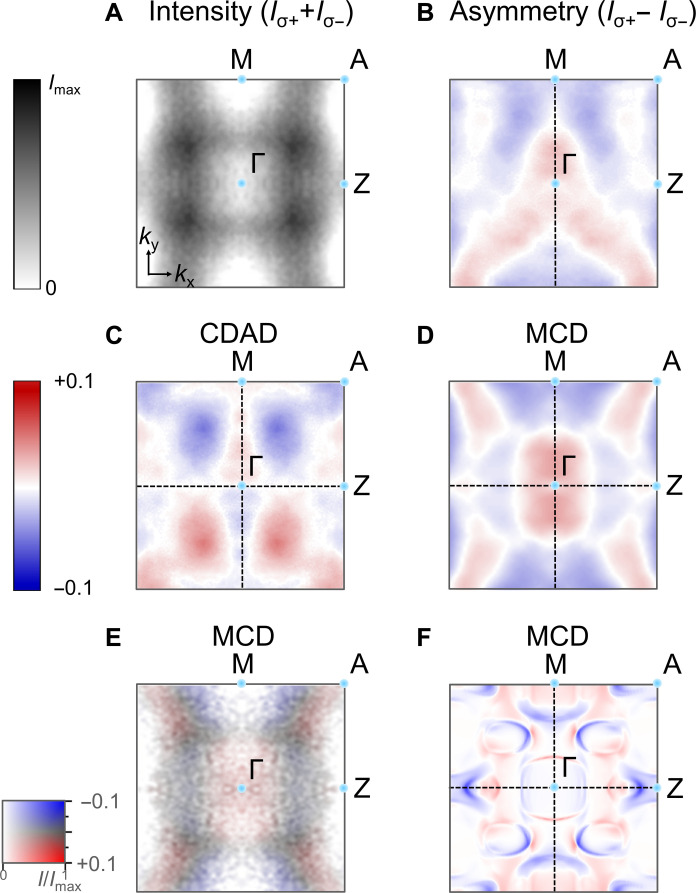
Experimental photoemission intensities and asymmetries at a high-symmetry Fermi surface section are compared to calculated data. (**A**) Constant energy map *I*(*E_F_*, *k_x_*, *k_y_*) measured at *hν* = 380 eV at 70 K on the Γ-M-A-Z plane. The intensity has been averaged for circular left and right polarization. Here, dark is high intensity (see calibration on the left side). (**B**) Asymmetry of the constant energy map (see color calibration on the left side). (**C**) CDAD map obtained from the asymmetry in (B). (**D**) MCD map obtained from the asymmetry in (B). (**E**) Combined MCD and intensity map on a two-dimensional color scale (see calibration on the left side). (**F**) Calculated MCD map distribution (not convoluted with the intensity). The red-blue features in the Γ-M line are clear between (E) and (F). Features on the edges near Z are overestimated since they are calculated on vanishingly small intensity features.

In Fig. 3, we present the measured intensity and asymmetry at the Fermi energy in the Γ-M-A-Z plane. The asymmetry is shown in Fig. 3B, and the corresponding CDAD and MCD components are shown in Fig. 3 (C and D). The convolution of the MCD with the average intensity plots is shown in Fig. 3E. It reveals the relevant parts of the MCD spectra, because some seemingly prominent features in Fig. 3D have very low intensity and hence are not directly reliably measured. For comparison, Fig. 3F shows the theoretical MCD (not convoluted with the intensity) at the Fermi energy.

We have confirmed the magnetic origin of the observed *A*_MCD_ spectra by repeating the experiment after rotating the sample around the surface normal by 180°, which effectively rotates the magnetic order. Domain sizes are likely larger than the footprint of the light beam, because driving forces for domain creation, such as stray field energy in ferromagnets and magnetostriction in multiaxial antiferromagnets, are absent in our uniaxial RuO_2_ films. Antiferromagnetic domains have been observed in the uniaxial antiferromagnet MnF_2_ using neutron diffraction and explained by the linear piezomagnetic effect in MnF_2_ that changes sign with the Néel vector (*55*). However, the piezomagnetic effect is very small and hence the domain sizes found in MnF_2_ are on a millimeter scale.

We present the experimental results and the corresponding theoretical calculations for both orientations in the Supplementary Materials (see figs. S3 and S4). The distribution of *A*_CDAD_ is similar to the results for the nonrotated sample. This can be expected because the (*k_x_*,*k_z_*) plane represents a crystal mirror plane. In contrast, *A*_MCD_ reversed its sign as expected. The spectra do not match exactly since due to the experimental setup limitations the area illuminated is not exactly the same, but even at this semiquantitative level, the conclusion remains the same. This result also confirms that the geometry of the experiment points to a spin quantization axis along *k_x_*, corresponding to the *c* axis of the RuO_2_ crystal structure.

We have further confirmed the results by performing ultraviolet excitation experiments with a photon energy of 6.4 eV using an infrared fiber laser with quadrupled photon energy. The results are restricted to a field of view limited to an area near the zone center (see fig. S7B). We present the circular dichroism results obtained for 6.4-eV photon energy in fig. S8. The asymmetries and decomposition in CDAD and MCD are calculated in the same way as for the soft x-ray results. The ultraviolet excitation results are fully consistent with the x-ray results depicted above.

In addition, as a control test, we performed the ultraviolet excitation photoemission experiment with the *c* axis oriented perpendicular to the incident light beam (see fig. S8, F to J). In this case, *A*_MCD_ (fig. S8J) vanishes within error limits. This observation indicates that the spin axis points perpendicular to the light polarization vector and hence parallel to the *c* axis [001], in agreement with the results obtained with the soft x-ray excitation.

**Fig. 4.**
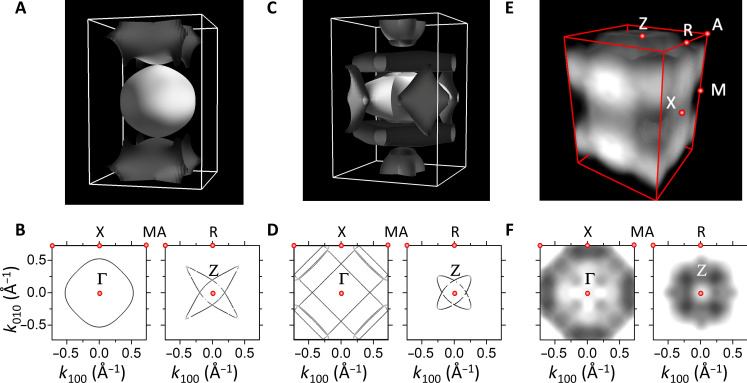
The calculated three-dimensional Fermi surface for the paramagnetic and altermagnetic case is compared to the measured Fermi surface. (**A**) Calculated Fermi surface for paramagnetic RuO_2_. (**B**) Fermi surface cut for the plane perpendicular to the *c* axis at the Γ-X-M plane and the Z-A-R plane for the paramagnetic phase. (**C**) Calculated Fermi surface for the collinear magnetically ordered RuO_2_. (**D**) Fermi surface cut for altermagnetic phase in the same planes as in (B). (**E**) Fermi surface obtained experimentally through a topographic mapping of the three-dimensional Brillouin zone (details in the Supplementary Materials). (**F**) Photoelectron intensity at the Fermi energy for planes as in (B).

To verify the altermagnetic phase of RuO_2_, we can compare the experimental results with key theoretically predicted features for the paramagnetic versus the altermagnetic phase. The theoretical calculations for the paramagnetic phase are shown in Fig. 4A, together with the Fermi surface cuts for the plane perpendicular to the *c* axis [110] at the Γ-X-M plane and the Z-A-R plane in Fig. 3B. We show the corresponding Fermi surface and cuts for the altermagnetic phase in Fig. 3 (C and D). The comparison to experiment is obtained by a tomographic mapping of the three-dimensional Brillouin zone (Fig. 1B), obtained by varying the photon energy in the range of 560 to 660 eV. According to Eq. 1, this variation results in *k_z_* = *k*_[110]_ values ranging from 5*G*_[110]_ to 5.5*G*_[110]_, i.e., from the center to the rim of a Brillouin zone (see fig. S5B). Here, *G*_[110]_ is the magnitude of the [110] reciprocal lattice vector. Exploiting the translational symmetry in momentum space, the intensity distributions *I*(*E_B_*, *k_x_*, *k_y_*, *k_z_*) map a complete Brillouin zone.

The Fermi energy intensity distribution shown along the same cuts and the overall shape of the reconstructed Fermi surface in Fig. 4 (E and F) match directly with the theoretical calculations of the collinear compensated altermagnetic phase in Fig. 3 (C and D). The altermagnetic phase shows a Brillouin zone crossing between Γ and M that is absent in the paramagnetic phase.

## DISCUSSION

We have experimentally established the key signature of the recently predicted (*13*, *14*) altermagnetic phase by directly detecting Tsymmetry breaking in the band structure of the collinear compensated magnet RuO_2_. Supported by ab initio calculations, our experimental results underpin on the microscopic electronic structure level the recently reported unconventional macroscopic responses, namely, the anomalous Hall and spin-polarized currents accompanied by vanishing magnetization (*23*, *30*–*33*), in this workhorse altermagnetic material. In general, our results microscopically establish the grounds for the exploration and exploitation of envisaged (*13*, *14*) phenomena and functionalities based on the altermagnetic Tsymmetry breaking that are beyond the reach of the conventional magnetic phases in fields ranging from spintronics, ultrafast magnetism, magnetoelectrics, and magnonics to topological matter and superconductivity.

## MATERIALS AND METHODS

We have grown epitaxial RuO_2_(110) films with a thickness of 34 nm by pulsed laser deposition on TiO_2_(110) substrates that were heated during deposition to 400°C. The samples show no detectable remanent magnetization, consistent with the earlier magnetometry studies of analogous RuO_2_/TiO_2_ thin films (*30*). For growth and sample characterization details, see the Supplementary Materials.

For the photoemission measurements, photoelectrons were excited by circularly polarized soft x-rays (beamline P04, PETRA III, DESY, Hamburg). For these experiments, we used the time-of-flight momentum microscope installed at the open port I of the beamline P04 with an energy resolution of 60 meV at a sample temperature of 70 K. In addition, circularly polarized ultraviolet light by a pulsed laser (6.4 eV, 80-MHz repetition rate, APE GmbH) was used. The photoemission experiments with laser excitation have been conducted using a time-of-flight momentum microscope (Surface Concept GmbH) with the resolution set to 40 meV (*48*) and at 20 K.

The circular dichroism photoemission experiments described below have been performed with the incidence angle of the photon beam at 22° with respect to the sample surface, and the azimuthal orientation of the sample has been adjusted so that the photon incidence plane coincides with the easy spin axis of RuO_2_, i.e., the [001] *c* axis (*18**,* *30**,* *44*).

The coordinate system for the photoelectron momentum (*k_x_*, *k_y_*, *k_z_*) is set to *k_z_* along the crystallographic [110] direction, i.e., surface normal, *k_x_* along [001] and *k_y_* along $\left[1\bar{1}0\right]$ in-plane directions, respectively. A sketch of the experimental geometry is shown in Fig. 1E.

## Supplementary Materials

### This PDF file includes:

Supplementary Text Figs. S1 to S9 References
