# Supplementary material for: Observation of time-reversal symmetry breaking in the band structure of altermagnetic RuO2
Source: Sci Adv. 2024 Jan 31;10(5):eadj4883. doi: 10.1126/sciadv.adj4883 (PMC10830110; doi:10.1126/sciadv.adj4883)
Supplement: Supplementary file 1 — Supplementary Text Figs. S1 to S9 References [file sciadv.adj4883_sm.pdf]

Science Advances
AAAS

Supplementary Materials for

Observation of time-reversal symmetry breaking in the band structure of altermagnetic RuO_2

Olena Fedchenko *et al.*

Corresponding author: Hans-Joachim Elmers, elmers@uni-mainz.de

Sci. Adv. **10**, eadj4883 (2024)
DOI: 10.1126/sciadv.adj4883

This PDF file includes:

Supplementary Text
Figs. S1 to S9
References

SUPPLEMENTARY MATERIAL

Sample preparation and characterization

Epitaxial $RuO_2(110)$ films with a thickness of 34 nm were grown by pulsed laser deposition on $TiO_2(110)$ substrates that were heated during deposition to 400 ^{o}C. The oxygen pressure was 0.02 mbar and typical growth rates were 1.9 nm/min with the KrF excimer laser running at 10 Hz and 150 mJ pulse energy. Samples were structurally characterized by X-ray diffraction, X-ray reflectometry and in-situ reflective high energy electron diffraction (RHEED). From X-ray reflectometry we find that 34 nm samples have a surface roughness smaller than 0.3 nm, while the width of the out-of plane scattering peak in 2Θ indicates a somewhat reduced coherent scattering volume of 22 nm. The existence of the RHEED scattering pattern shows that crystallinity persists up to the surface. The full width at half maximum of the rocking curve is 1.0^o indicating a good alignment of the surface normal. Φ scans reveal the epitaxial growth and show a two fold symmetry of (200) peaks aligned with that from the substrate. The width of the peaks in Φ is 2°. From the determined lattice constants we conclude on a compressive strain of -1.4% along c-axis and a tensile strain of 0.4% along a-axis.

Samples were transported from the deposition chamber to the photoemission experiment using an ultra-high vacuum suitcase.

We have performed magnetization measurements using SQUID (MPMS II) magnetometry. The magnetic moment of the samples was measured at 70 K in a magnetic field applied parallel to the [001] direction with field $\mu_0 H$ decreasing from 4 T to -4 T (see Fig. S1). The diamagnetic contribution of the substrate yields a straight line above 1 T. The data was then corrected for the diamagnetic contribution and rescaled to the number of Bohr magnetons μ_B per Ru atom. At saturation there is a small saturation magnetization of about μ_B per Ru atom. However, for zero field, at which the ARPES-MCD was measured, the remnant contribution is vanishing within error limits. The upper limit for the remnant magnetization is estimated to 0.001 μ_B per Ru atom. Hysteresis loops measured for a thinner (10 nm) sample also show no remnant magnetization.

In addition, we have used magneto-optical Kerr (MOKE) microscopy to characterize possible domains. We did not observe any contrast, reenforcing again that the Ru moments are collinear and pointing along the [001] easy axis, for which the Hall vector and any observable MOKE is excluded by symmetry.

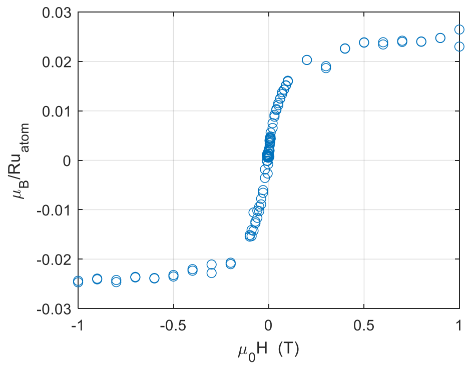

FIG. S1. Magnetization loop measured for the magnetic field applied parallel to the [001] direction. The sample is identical to the sample that has been used for the soft X-ray photoemission experiment.

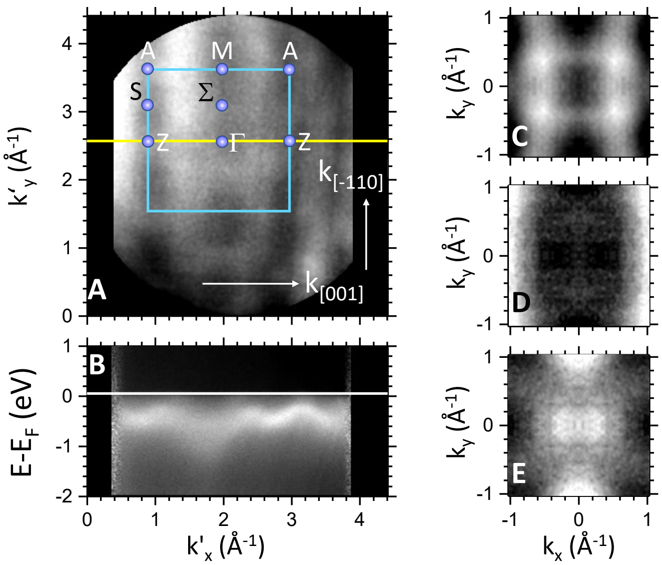

FIG. S2. Photoemission intensities are depicted as sections from the 3D data array. **A** Constant energy map $I(E_F, k_x, k_y)$ measured at $h\nu = 380$ eV at 70 K. The intensity has been averaged for circular left and right polarization. The photon beam impinges from the right parallel to k_x. The blue square indicates the Brillouin zone boundary. **B** Intensity map $I(E_B, k_x)$ revealing the energy dispersion of bands along the profile indicated by the horizontal yellow line in **A**. **C**-**E** Symmetrized constant energy maps covering one Brillouin zone at binding energies **C** $E_B = 0$, **D** 0.5, and **E** 1.0 eV at $h\nu = 660$ eV.

Details of soft X-ray excitation

As we have described in the main text, using soft X-ray excitation measures the photoelectron intensity distribution $I(E_B, k_x, k_y, k_z)$. Considering the energy conservation and the role of reciprocal lattice vectors in order to obey momentum conservation, for a given photon energy $h\nu$ and binding energy $E_B = E - E_F$, the final photoelectron states are located on a spherical shell with radius (for units Å^{-1} and eV)

$$k_f = 0.512\sqrt{h\nu - E_B + V_0^*}. \tag{S1}$$

Here, we assume free-electron like final states, whose final-state energies inside the material are determined by the inner potential $V_0^* \approx 10$ eV referenced to the Fermi energy. The transferred photon momentum leads to a rigid shift of the final state sphere by the vector with absolute value $k_{h\nu} = 2\pi\nu/c$ along the photon beam [48].

The kinetic energy of the emitted photoelectrons is recorded by their time of flight. The Fermi edge serves as reference for $E_B = 0$. The pattern observed on the detector represents the photoelectron intensity distribution as a function of the transversal momentum $k_{f,||}$.

We next discuss the photoelectron intensity distributions averaged for circular left and right polarization. Fig. S2**A,B** shows results recorded at a photon energy of 380 eV corresponding to final states in the 5th repeated BZ along the direction perpendicular to the surface (k_z). The results are shown as raw data divided by the detector response function. The constant energy map [Fig. S2**A**] at the Fermi level reveals the two-fold symmetry of the $RuO_2(110)$ surface. The cut along the high symmetry direction $\Gamma - Z$ indicates the energy dispersion of the valence band $E_B(k_x)$ [Fig. S2**B**]. We observe a homogeneous background intensity and the maximum photoelectron intensity of direct transitions amounts to 20% of the background intensity, originating from quasi-inelastic scattering. The electron bands appear broadened both on the energy and momentum scale, which cannot be explained by the finite energy and momentum resolution of the instrument. Instead, we attribute the broadening to the electron correlation, which is expected to be large in oxides [46].

The symmetrized constant energy maps centered at Γ in the range of $E_B = 0-1$ eV [Fig. S2**C**-**E**] have been acquired for $h\nu = 660$ eV and cover one Brillouin zone. The maps agree with previously published results [47] on RuO_2 single crystals obtained with a photon energy of 131 eV, except for the localized high intensity at the crossing points of horizontal and vertical lines, which may stem from surface states. Hence, we attribute the observed photoelectron intensity distribution to bulk states of RuO_2.

To further verify the results of the circular dichroism observations, we show the intensity maps at the Fermi level for circular left and right polarization [Fig. S3**A,B**]. Here, as compared to the main text, the asymmetry, CDAD, and

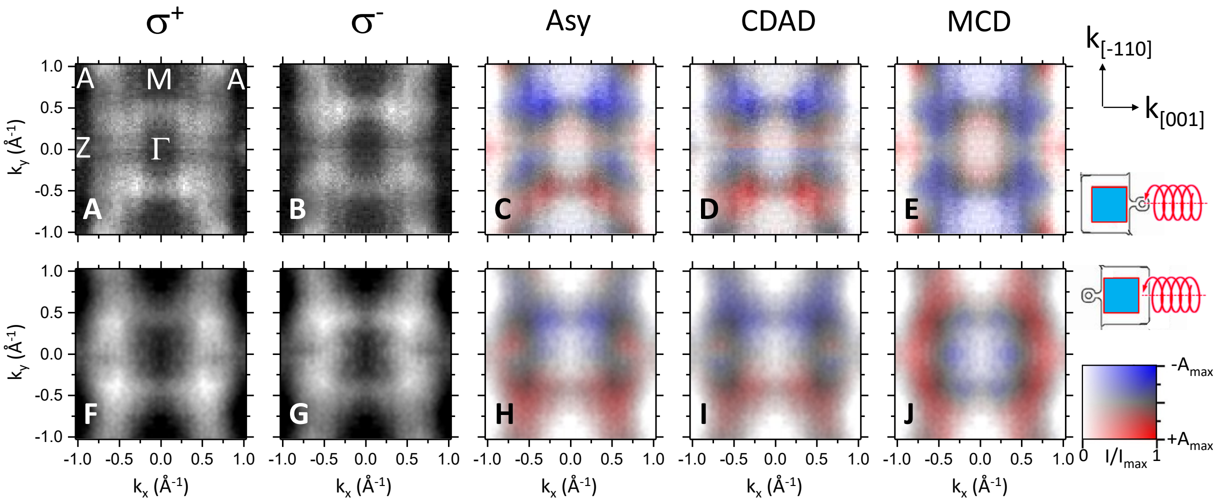

FIG. S3. Photoemission intensities and asymmetries measured for opposite Néel vector directions show opposite MCD. **A,B** Photoelectron intensity maps at the Fermi energy obtained with right (σ^+) and left (σ^-) circularly polarized light. **C** Asymmetry and averaged intensity depicted in a combined color scale. **D** CDAD and intensity in the same color scale. **E** MCD and intensity depicted in a combined color scale. **F-J** Similar data for the sample being rotated by 180 degrees as indicated by the sketches on the right.

MCD are presented convoluted with the averaged intensity maps [Fig. S3**C-E** and **H-J**]. As with the results presented in Fig. 2, the asymmetry is decomposed in the CDAD and MCD components. The result shown in Fig. S3**D** reveals predominantly negative values for positive k_y except for a reversed sign close to $k_y = 0$. The maximum experimental values amount to $\pm 5.4\%$. After subtracting the homogeneous background intensity, the CDAD for direct transitions is $\pm 27\%$ and its size is thus in the range of previously reported values [56]. For non-magnetic systems A_{CDAD} is the only possible circular dichroism mechanism except for crystal structures with natural chirality, where effects are extremely small. On the other hand, CDAD is ubiquitous in photoemission because it is a pure orbital effect and does not require spin-orbit interaction and heavy elements.

The MCD data show a negative asymmetry for the vertical stripes parallel to Γ-M and positive values for the horizontal strips parallel to Γ-Z. Along the M-Γ-M path in Fig. S3**E**, A_{MCD} is negative near the M-points and positive near the Γ point, in good agreement with the results shown in Fig. 2**C** for the Fermi level. The maximum values of A_{MCD} are $\pm 15\%$. A_{MCD} is thus remarkably large compared to values of a few percent reported for $3d$ metallic ferromagnets [35].

To confirm that the observed A_{MCD} is connected to the magnetic order of the sample, we rotated the sample around the surface normal by 180 degrees and repeated the photoemission experiment, albeit not in the same exact spot given the experimental limitations of the set-up. Corresponding results are shown in Figs. S3**F-J**. The distribution of A_{CDAD} is similar to the results for the non-rotated sample. This can be expected because the (k_x,k_z) plane represents a crystal mirror plane. In contrast, A_{MCD} has reversed its sign. The reversal of the sign of the MCD data further confirms that the observations and those in Fig. 2 are a directly observation of the broken time-reversal symmetry of the antiferromagnetic sample.

In Fig. S4 we present the theory calculations corresponding to the experiment and the spin-resolved bands presented in S4**E** and **J**. Here the color plots are not convoluted with the intensity for easier visualization. Fig. S4**A-E** corresponds to the experimental set-up of S3**A-E** and Fig. S4**F-J** corresponds to the experimental set-up of S3**F-J**.

We present the scan of the perpendicular momentum by photon energy in Fig. S5. This topographic mapping is made by varying the photon energy in the range of 560 - 660 eV. According to Eq. S1 this variation results in $k_z = k_{[110]}$ values ranging from $5G_{[110]}$ to $5.5G_{[110]}$, i.e. from the center to the rim of a Brillouin zone [see Fig. S5**B**]. Similarly, corresponding asymmetry maps were determined. Exploiting the translational symmetry in momentum space, the intensity distributions $I(E_B, k_x, k_y, k_z)$ map a complete Brillouin zone. This topographic map and cuts are the ones used in Fig. 4.

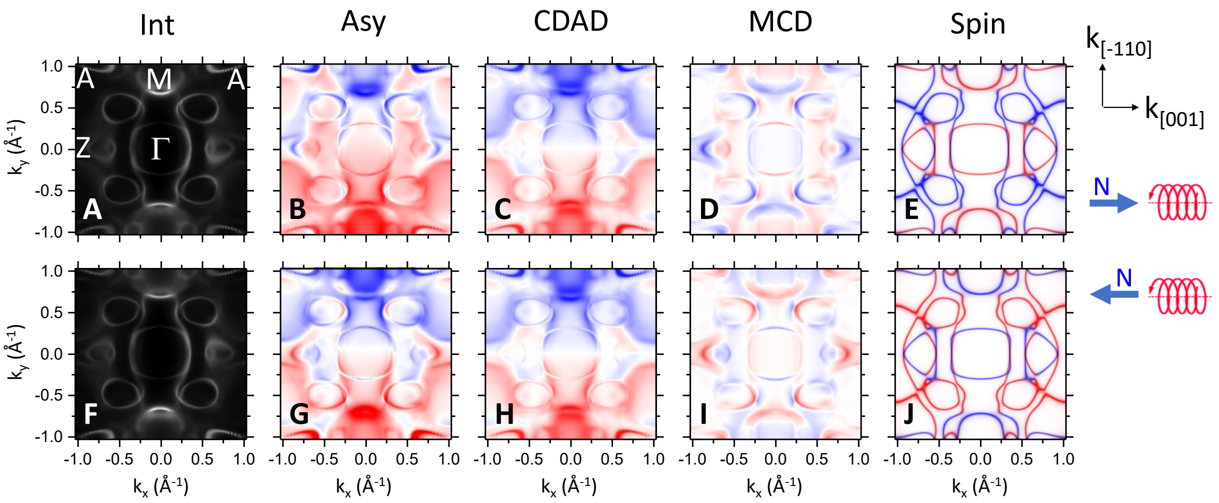

FIG. S4. Photoemission intensities and asymmetries calculated for opposite Néel vector directions show opposite MCD. **A** Calculation of photoelectron intensity maps at the Fermi energy. **B** Asymmetry depicted in a color scale. **C** CDAD in the same color scale. **D** MCD in the same color scale. **E** Spin-resolved bands. **F-J** Similar data for the sample being rotated by 180 degrees as indicated by the sketches on the right.

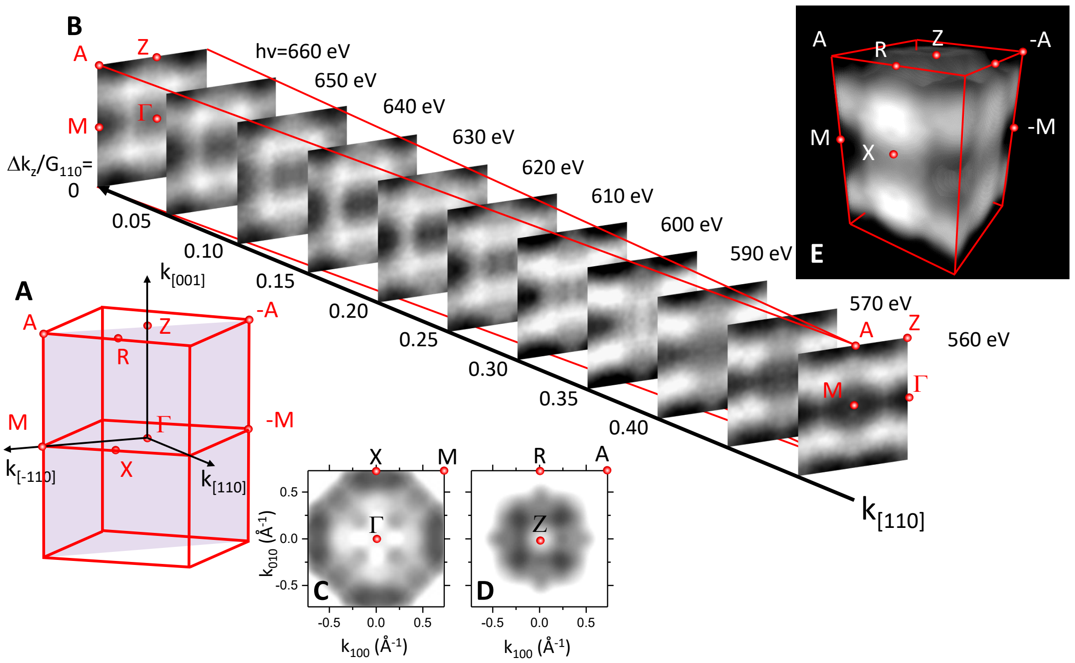

FIG. S5. The 3D Fermi surface is constructed from photoemission data measured for a sequence of photon energies. **A** Sketch of the Brillouin zone of the RuO_2 rutile structure in momentum space. **B** Series of photoelectron intensity maps at the Fermi level for indicated photon energies. **C**-**D** Photoelectron intensity at the Fermi surface for planes perpendicular to the c-axis [110] at $k_z = 0$ and $0.5G_{110}$. **E** Three-dimensional Fermi surface.

Comparison of calculated and experimental photoemission intensities

Figure S6 compares the calculated photoemission intensity (left color scale) with the calculated initial state bulk band structure (thin yellow lines) for the central Brillouin zone **B**, also shown in the main text, and an adjacent Brillouin zone **A**. The initial state bulk band structure shows only small differences due to the slightly varied perpendicular momentum. In contrast, the calculated photoemission intensity shows a significant variation of some bands

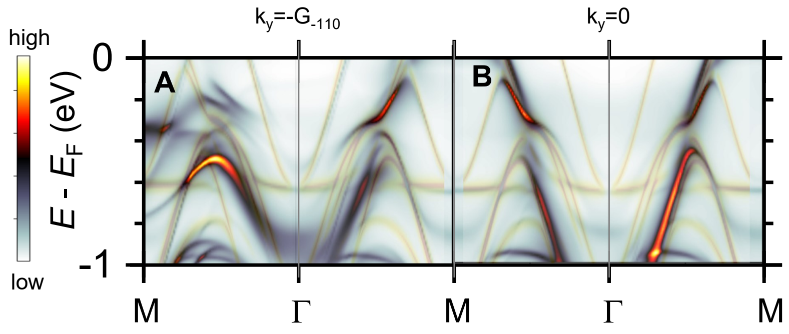

FIG. S6. Calculated photoemission intensity (left color scale) are presented in the range of two adjacent Brillouin zones. **A** M-Γ-M section at $k_y = -G_{-110}$. **B** M-Γ-M section at $k_y = 0$, also shown in Fig. 2**E**. Overlaid is the corresponding initial state Bloch spectral function (thin yellow lines) calculated with the same parameters.

that have very high photoemission intensity. We propose tentatively that the noted high intensities, without a distinct counterpart in the initial band structure, might be linked to surface resonance [57]. This interpretation relies on multiple model calculations. Upon deactivating surface barrier contributions to the photocurrent, several surface-related bands become seemingly evident. However, they still fail to entirely elucidate the pronounced intensity variation in the regions at a binding energy of -0.5 eV. In our case, the imperfect surface of the as grown films prohibits the observation of the calculated high intensity structures. Except for these high photoemission intensities, the calculated photoemission intensity and the initial state bands correspond to each other. The exchange splitting of the two spin-polarized bands at the Γ- point and binding energy 0.6 eV corresponds to the experimental photoemission intensity considering a broadening due to the averaging along k_x.

Results with ultraviolet excitation

As compared to the previous results, photoelectron excitation with a photon energy of 6.4 eV using an infrared fibre laser with quadrupled photon energy is favourable with respect to photon intensity. Yet, the small photon energy limits the detectable parallel momentum to $k_{||} < 0.6$ Å^{-1} [see Fig. S7**A**]. Assuming an excitation into free-electron like final states [58], Eq. S1 results in a cut close to the center of the second repeated Brillouin zone (Γ) in momentum space [see Fig. S7**B**]. At increased binding energy, we still observe the two pairs of horizontal and vertical bands, which we identify with the band features seen with soft X-ray excitation. The dispersion maps [Figs. S7**C,D**] reveal a maximum binding energy of the parabolic band at Γ of 0.6 eV, in reasonable agreement with results obtained with soft X-ray excitation. When comparing band dispersions at different photon energies, the different cuts in the three-dimensional momentum-space and a possible variation of the effective mass must be considered.

The circular dichroism results obtained for 6.4 eV photon energy are shown in Fig. S8. The asymmetries are calculated in the same way as for the soft X-ray results. In this case the azimuthal orientation could not be varied in-situ and hence we compare to similarly prepared samples with the c-axis oriented parallel and perpendicular to the incident light beam. For the case of parallel orientation [Fig. S8**A-E**] $A(k_x, k_y)$ shows the expected antisymmetric behavior with respect to the $\Gamma - Z$ symmetry axis, originating from non-relativistic CDAD [49,50]. By calculating $2A_{\mathrm{CDAD}}(k_x, k_y) = A(k_x, k_y) - A(k_x, -k_y)$ we separate the CDAD asymmetry as shown in Fig. S3**C-D** and **H-I**. The CDAD shows the same antisymmetry as in the soft X-ray case with the characteristic feature of a sign change at the line $k_y = 0$. This line corresponds to the coplanar geometry of crystal mirror plane and photon incidence plane. The maximum experimental values amount to $\pm 26\%$.

The calculation of $2A_{\mathrm{MCD}} = A(k_x, k_y) + A(k_x, -k_y)$ results in the magnetic contribution to the circular dichroism [Fig. S8**E**]. We observe a positive asymmetry for the vertical stripes parallel to Γ-M and negative values for the horizontal strips parallel to Γ-Z. The maximum values of A_{MCD} are $\pm 13\%$. A_{MCD} observed with 6.4 eV excitation thus confirms the non-vanishing magnetic circular dichroism observed for soft X-ray excitation.

A similar photoemission experiment with the c-axis orientated perpendicular to the incident light beam serves as a control test [Fig. S8**F-J**]. In this case, A_{CDAD} shows a left-right asymmetry with respect to the Γ-M axis [Fig. S8**I**]. A_{MCD} [Fig. S8**J**] vanishes within error limits. This observation indicates that the magnetization axis points perpendicular to the light polarization vector and hence parallel to the c-axis [001], in perfect agreement with the results obtained with soft X-ray excitation.

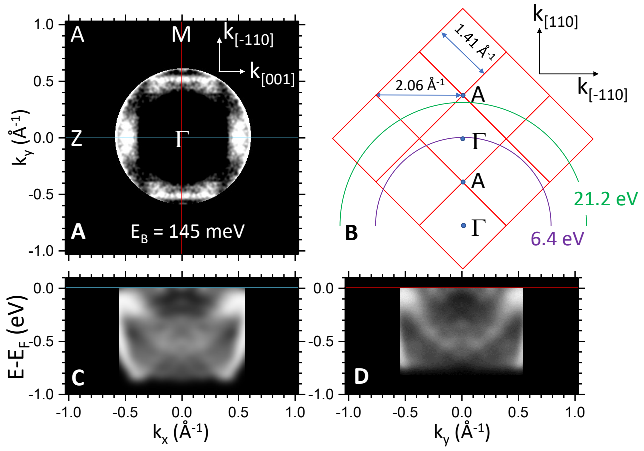

FIG. S7. Photoemission intensities are measured with an excitation photon energy of 6.4 eV. **A** Polarization-averaged photoelectron intensity map at a binding energy of 145 meV for excitation with a photon energy of 6.4 eV. The photon beam impinges from the right along the in-plane [001] direction of the $RuO_2(110)$ film. **B** Sketch of the repeated Brillouin zone scheme in a plane perpendicular to the [001] axis. Green and violet half circles indicate the free-electron final state momenta for photon energies 21.2 eV and 6.4 eV, respectively. **C** Photoelectron intensity map in the E_B vs. k_x plane revealing the dispersion close to the Γ-Z direction. **D** Same, but along the Γ-M (k_y) direction, corresponding to the violet half-circle in **B**.

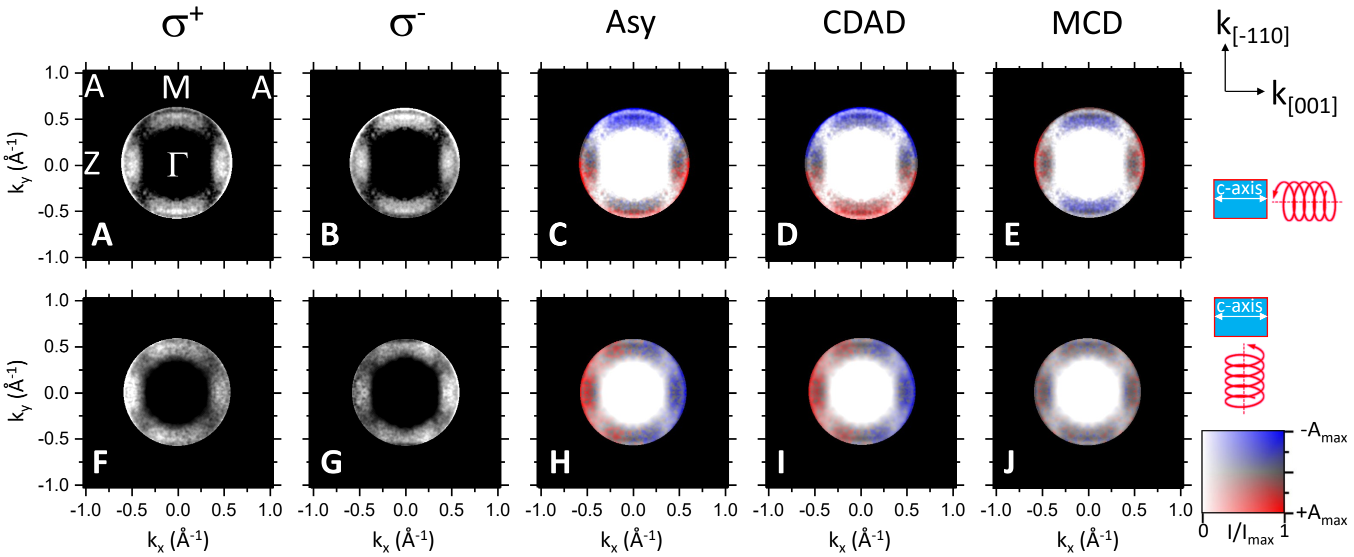

FIG. S8. Photoemission intensities and asymmetries measured for perpendicularly oriented Néel vector directions confirm the origin of the MCD to be the sublattice magnetization direction. **A,B** Photoelectron intensity maps at a binding energy of 182 meV obtained with 6.4 eV photon energy and circularly right (σ^+) and left (σ^-) polarized light. **C** Asymmetry and averaged intensity plotted in a combined color scale. **D** CDAD and intensity depicted in the same color scale. **E** Same, but for the corresponding MCD and intensity. **F-J** Similar data for 90 degrees rotated sample as indicated by the sketches on the right.

Ab initio based calculation details

We have calculated the ground state electronic structure of RuO_2 in P42/mnm (Space group:136) symmetry using the optimized lattice parameter (a = b = 4.5331, c = 3.1241Å) [18]. These calculations were carried out using spin-polarized relativistic Korringa-Kohn-Rostoker (SPRKKR) Green's function method in the atomic sphere approximation (ASA), within the rotationally invariant GGA+U scheme as implemented in the SPRKKR formalism [51,59]. The screened on-site Coulomb interaction U and exchange interaction J of Ru are set to 2.00 eV and 0.70 eV, respec-

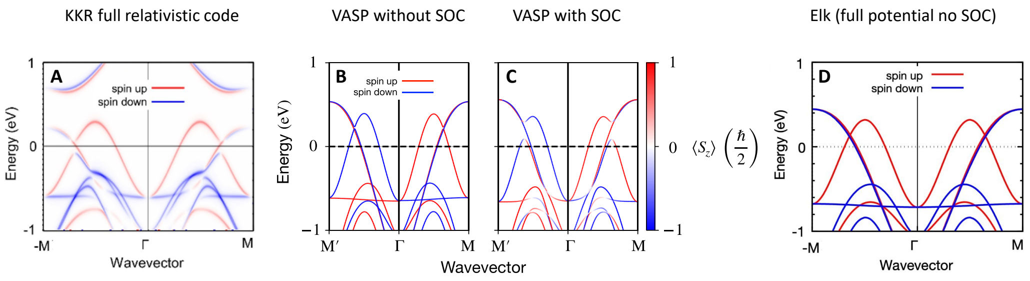

FIG. S9. Comparison of calculations using different codes show similar results. Calculated band structure using **A** the fully relativistic KKR code, **B** VASP without spin-orbit coupling, **C** VASP with spin-orbit coupling, and **D** Elk without spin-orbit coupling. The VASP results are shown along the M' -Γ -M lines, rather than the -M-Γ-M, to illustrate the 4-fold symmetry of the splitting. M' is oriented at a 90° relative to M.

tively. The angular momentum expansion up to $l_{\max} = 4$ has been used for each atom on a 22 x 22 x 32 k-point grid. The energy convergence criterion has been set to 10^{-5} Ry. Lloyd's formula has been employed for accurate determination of the Fermi level [60]. The ab initio photoemission calculations of $RuO_2(110)$ were performed within the one-step model of photoemission in the spin-density-matrix formulation as implemented in the SPRKKR package [52], taking into account all geometry and light-induced effects of the photoemission process for the actual experiment including photoelectron angular distribution, matrix elements and final states constructed as the time-reversed LEED states. The final-state damping was described via constant $V_i = 2.7$ eV set to simulate finite innelastic mean free path.

In Fig. S9 we compare the ground state band structure using the SPRKKR code in the atomic sphere approximation (ASA), the pseudo-potential based code VASP uses U-J=1.3 eV (with and without spin-orbit coupling), and the full potential based code Elk, with values as in the SPRKKR code, U=2 eV and J=0.7 eV.

Approximation of combined CDAD and MCD

The circular dichroism in the angular distribution (CDAD) asymmetry in the absence of magnetic order can be defined as $A_{\mathrm{CDAD}} = (I_+ - I_-)/(I_+ + I_-)$ [49]. The asymmetry means that the photoemission intensity depends on the circular polarization of the incident x-ray radiation according to $I_+ = I_0(1 + c_{\mathrm{CDAD}})$ and $I_- = I_0(1 - c_{\mathrm{CDAD}})$. The angular dependent parameter c_{CDAD} can be described as $c_{\mathrm{CDAD}} = c_{\mathrm{CDAD},0}(\hat{n} \times \vec{P}) \cdot \hat{k}_y$, where $\hat{n}$ is the unit vector along the surface normal, $\vec{P}$ the polarization of the incident x-ray radiation and $\hat{k}_y$ the unit vector along the k_y momentum direction perpendicular to the plane of incidence [see Fig. 1**E**]. The representation of c_{CDAD} satisfies the time-reversal symmetry for the non-relativistic CDAD [56]. In the following, we replace $\vec{P}$ with its planar projection $P = \pm 1$ and include the correction factor in the parameter c_{CDAD}. We can then write the photoemission intensities for circular left and right polarization as $I_i = I_0(1 + ic_{\mathrm{CDAD},0})$ with $i = P\,\mathrm{sign}(k_y) = \pm 1$. Written as an index, i is defined as $i = +$ or $i = -$, respectively. Note that because of the symmetry condition $A_{\mathrm{CDAD}} = 0$ for $k_y = 0$ and therefore the angular distribution is essential to the CDAD.

In a similar way, the magnetic circular dichroism in the angular distribution (MCDAD) [or in the short version magnetic circular dichroism (MCD)] in the absence of CDAD is defined as $A_{\mathrm{MCD}} = (I^+ - I^-)/(I^+ + I^-)$ [36]. The photoemission intensity depends on the relative orientation of the magnetization vector and the polarization of the incident x-ray beam. We stress this definition by replacing the magnetization vector by the N'eel vector $\vec{N}$. The photoemission intensity is then given by $I^+ = I_0(1 + c_{\mathrm{MCD}})$ and $I^- = I_0(1 - c_{\mathrm{MCD}})$. The angular dependent parameter c_{MCD} can be described as $c_{\mathrm{MCD}} = c_{\mathrm{MCD},0}\vec{P} \cdot \hat{N}$, where $\vec{P}$ is the polarization of the incident x-ray along the Néel vector [see Fig. 1**E**]. The correction factor for the planar projection of $\hat{N}$ is included in c_{MCD}. We can then write the photoemission intensities as $I^k = I_0(1 + kc_{\mathrm{MCD},0})$ with $k = P\hat{N} = \pm 1$. Written as an index, k is defined as $k = +$ or $k = -$, respectively. In this case A_{MCD} is not necessarily zero for $k_y = 0$ and therefore the angular distribution is not essential to the MCDAD. This is the reason that we can write MCD instead of MCDAD.

Now we turn to the case of the presence of both types of asymmetries. In general, one can measure four independent photoemission intensities I_i^k, with $k = +, -$ and $i = +, -$. The asymmetries are linear combinations of these four intensities. The MCD asymmetry for positive polarization is calculated as the difference in the averages over k_y and $-k_y$ for positive Néel vector and negative Néel vector, respectively. More precisely, one obtains:

$$A_{\text{MCD}} = \frac{(I_+^+ + I_-^+) - (I_+^- + I_-^-)}{(I_+^+ + I_-^+) + (I_+^- + I_-^-)}. \tag{S2}$$

For negative polarization, one obtains the same value but with opposite sign.

The CDAD asymmetry for positive k_y is calculated as the difference in the averages over positive and negative Néel vector for positive polarization and negative polarization, respectively:

$$A_{\text{CDAD}} = \frac{(I_+^+ + I_+^-) - (I_-^+ + I_-^-)}{(I_+^+ + I_+^-) + (I_-^+ + I_-^-)}. \tag{S3}$$

As it is clear from the above equations, experimentally defining the MCD and the CDAD, that the total asymmetry is not the sum of MCD and CDAD. However, for small asymmetries, we can approximate all intensities values in the denominator by I_0. The error is negligible for asymmetries in the range of a few percent. In this case, the MCD asymmetry reads as follows:

$$A_{\text{MCD}} = \frac{1}{4I_0}(I_+^+ + I_-^+ - I_+^- - I_-^-). \tag{S4}$$

For the CDAD we obtain:

$$A_{\text{CDAD}} = \frac{1}{4I_0}(I_+^+ + I_+^- - I_-^+ - I_-^-). \tag{S5}$$

The asymmetry A determined from the experimental data and defined as $A = (I_+ - I_-)/(I_+ + I_-)$, where I_P is the intensity for positive and negative polarization, can be approximated as $A = \frac{1}{2I_0}(I_+^+ - I_-^-)$ for $k_y \geq 0$ and $A = \frac{1}{2I_0}(I_-^+ - I_+^-)$ for $k_y < 0$. In the main text the magnetic circular dichroism is calculated as:

$$A_{\text{MCD}} = [A(k_x, k_y) + A(k_x, -k_y)]/2 = \frac{1}{4I_0}[(I_+^+ - I_-^-) + (I_-^+ - I_+^-)]. \tag{S6}$$

Comparison with Eq. S4 shows that Eq. S4 and Eq. S6 are equivalent. A similar calculation for the asymmetry A_{CDAD} in the main text leads to

$$A_{\text{CDAD}} = [A(k_x, k_y) - A(k_x, -k_y)]/2 = \frac{1}{4I_0}[(I_+^+ - I_-^-) - (I_-^+ - I_+^-)]. \tag{S7}$$

Comparison with Eq. S5 shows that the definition in the main text (Eq. S7) and the general results of Eq. S5 are equivalent.

REFERENCES AND NOTES

1. N. Nagaosa, J. Sinova, S. Onoda, A. H. MacDonald, N. P. Ong, Anomalous Hall effect. *Rev. Mod. Phys.* **82**, 1539 (2010).
2. Y. Tokura, K. Yasuda, A. Tsukazaki, Magnetic topological insulators. *Nat. Rev. Phys.* **1**, 126–143 (2019).
3. L. Šmejkal, A. H. MacDonald, J. Sinova, S. Nakatsuji, T. Jungwirth, Anomalous Hall antiferromagnets. *Nat. Rev. Mater.* **7**, 482–496 (2022).
4. C. Chappert, A. Fert, F. N. Van Dau, The emergence of spin electronics in data storage. *Nat. Mater.* **6**, 813–823 (2007).
5. D. C. Ralph, M. D. Stiles, Spin transfer torques. *J. Magn. Magn. Mater.* **320**, 1190–1216 (2008).
6. S. D. Bader, S. Parkin, Spintronics. *Annu. Rev. Condens. Matter Phys.* **1**, 71–88 (2010).
7. S. Bhatti, R. Sbiaa, A. Hirohata, H. Ohno, S. Fukami, S. Piramanayagam, Spintronics based random access memory: A review. *Mater. Today* **20**, 530–548 (2017).
8. T. Jungwirth, X. Marti, P. Wadley, J. Wunderlich, Antiferromagnetic spintronics. *Nat. Nanotechnol.* **11**, 231–241 (2016).
9. A. Manchon, J. Železný, I. M. Miron, T. Jungwirth, J. Sinova, A. Thiaville, K. Garello, P. Gambardella, Current-induced spin-orbit torques in ferromagnetic and antiferromagnetic systems. *Rev. Mod. Phys.* **91**, 035004 (2019).
10. T. Jungwirth, J. Sinova, A. Manchon, X. Marti, J. Wunderlich, C. Felser, The multiple directions of antiferromagnetic spintronics. *Nat. Phys.* **14**, 200–203 (2018).
11. V. Baltz, A. Manchon, M. Tsoi, T. Moriyama, T. Ono, Y. Tserkovnyak, Antiferromagnetic spintronics. *Rev. Mod. Phys.* **90**, 015005 (2018).
12. A. V. Kimel, M. Li, Writing magnetic memory with ultrashort light pulses. *Nat. Rev. Mater.* **4**, 189–200 (2019).
13. L. Šmejkal, J. Sinova, T. Jungwirth, Beyond conventional ferromagnetism and antiferromagnetism: A phase with nonrelativistic spin and crystal rotation symmetry. *Phys. Rev. X* **12**, 031042 (2022).
14. L. Šmejkal, J. Sinova, T. Jungwirth, Emerging research landscape of altermagnetism. *Phys. Rev. X* **12**, 040501 (2022).
15. H.-J. Elmers, S. V. Chernov, S. W. D'Souza, S. P. Bommanaboyena, S. Y. Bodnar, K. Medjanik, S. Babenkov, O. Fedchenko, D. Vasilyev, S. Y. Agustsson, C. Schlueter, A. Gloskovskii, Y. Matveyev, V. N. Strocov, Y. Skourski, L. Šmejkal, J. Sinova, J. Minár, M. Kläui, G. Schönhense, M. Jourdan,

Néel vector induced manipulation of valence states in the collinear antiferromagnet Mn_2Au. *ACS Nano* **14**, 17554–17564 (2020).

16. I. E. Dzyaloshinkii, *J. Exptl. Theoret. Phys.* **37**, 881–882 (1959).
17. T. Moriya, Anisotropic superexchange interaction and weak ferromagnetism. *Phys. Rev.* **120**, 91–98 (1960).
18. L. Šmejkal, R. González-Hernández, T. Jungwirth, J. Sinova, Crystal time-reversal symmetry breaking and spontaneous Hall effect in collinear antiferromagnets. *Sci. Adv.* **6**, eaaz8809 2020.
19. K. Samanta, M. Ležaić, M. Merte, F. Freimuth, S. Blügel, Y. Mokrousov, Crystal Hall and crystal magneto-optical effect in thin films of $SrRuO_3$. *J. Appl. Phys.* **127**, 213904 (2020).
20. M. Naka, S. Hayami, H. Kusunose, Y. Yanagi, Y. Motome, H. Seo, Anomalous Hall effect in κ-type organic antiferromagnets. *Phys. Rev. B* **102**, 075112 (2020).
21. S. Hayami, H. Kusunose, Essential role of the anisotropic magnetic dipole in the anomalous Hall effect. *Phys. Rev. B* **103**, L180407 (2021).
22. I. I. Mazin, K. Koepernik, M. D. Johannes, R. González-Hernández, L. Šmejkal, Prediction of unconventional magnetism in doped $FeSb_2$. *Proc. Natl. Acad. Sci. U.S.A.* **118**, e2108924118 (2021).
23. R. D. Gonzalez Betancourt, J. Zubáč, R. Gonzalez-Hernandez, K. Geishendorf, Z. Šobáň, G. Springholz, K. Olejník, L. Šmejkal, J. Sinova, T. Jungwirth, S. T. B. Goennenwein, A. Thomas, H. Reichlová, J. Železný, D. Kriegner, Spontaneous anomalous hall effect arising from an unconventional compensated magnetic phase in a semiconductor. *Phys. Rev. Lett.* **130**, 036702 (2023).
24. M. Naka, Y. Motome, H. Seo, Anomalous Hall effect in antiferromagnetic perovskites. *Phys. Rev. B* **106**, 195149 (2022).
25. M. Naka, S. Hayami, H. Kusunose, Y. Yanagi, Y. Motome, H. Seo, Spin current generation in organic antiferromagnets. *Nat. Commun.* **10**, 4305 (2019).
26. R. González-Hernández, L. Šmejkal, K. Výborný, Y. Yahagi, J. Sinova, T. Jungwirth, J. Železný, Efficient electrical spin splitter based on nonrelativistic collinear antiferromagnetism. *Phys. Rev. Lett.* **126**, 127701 (2021).
27. M. Naka, Y. Motome, H. Seo, Perovskite as a spin current generator. *Phys. Rev. B* **103**, 125114 (2021).
28. H.-Y. Ma, M. Hu, N. Li, J. Liu, W. Yao, J.-F. Jia, J. Liu, Multifunctional antiferromagnetic materials with giant piezomagnetism and noncollinear spin current. *Nat. Commun.* **12**, 2846 (2021).
29. L. Šmejkal, A. B. Hellenes, R. González-Hernández, J. Sinova, T. Jungwirth, Giant and tunneling

magnetoresistance in unconventional collinear antiferromagnets with nonrelativistic spin-momentum coupling. *Phys. Rev. X* **12**, 011028 (2022).

30. Z. Feng, X. Zhou, L. Šmejkal, L. Wu, Z. Zhu, H. Guo, R. González-Hernández, X. Wang, H. Yan, P. Qin, X. Zhang, H. Wu, H. Chen, Z. Meng, L. Liu, Z. Xia, J. Sinova, T. Jungwirth, Z. Liu, An anomalous Hall effect in altermagnetic ruthenium dioxide. *Nat. Electron.* **5**, 735–743 (2022).
31. A. Bose, N. J. Schreiber, R. Jain, D.F. Shao, H. P. Nair, J. Sun, X. S. Zhang, D. A. Muller, E. Y. Tsymbal, D. G. Schlom, D. C. Ralph, Tilted spin current generated by the collinear antiferromagnet ruthenium dioxide. *Nat. Electron.* **5**, 267–274 (2022).
32. H. Bai, L. Han, X. Feng, Y. Zhou, R. Su, Q. Wang, L. Liao, W. Zhu, X. Chen, F. Pan, X. L. Fan, C. Song, Observation of spin splitting torque in a collinear antiferromagnet RuO_2. *Phys. Rev. Lett.* **128**, 197202 (2022).
33. S. Karube, T. Tanaka, D. Sugawara, N. Kadoguchi, M. Kohda, J. Nitta, Observation of spin-splitter torque in collinear antiferromagnetic RuO_2. *Phys. Rev. Lett.* **129**, 137201 (2022).
34. A. Hariki, T. Yamaguchi, D. Kriegner, K. W. Edmonds, P. Wadley, S. S. Dhesi, G. Springholz, L. Šmejkal, K. Výborný, T. Jungwirth, J. Kuneš, X-ray magnetic circular dichroism in altermagnetic α-MnTe. arXiv:2305.03588 [cond-mat.mtrl-sci] (2023).
35. C. M. Schneider, M. S. Hammond, P. Schuster, A. Cebollada, R. Miranda, J. Kirschner, Observation of magnetic circular dichroism in UV photoemission from fcc cobalt films. *Phys. Rev. B* **44**, 12066–12069 (1991).
36. J. Bansmann, C. Westphal, M. Getzlaff, F. Fegel, G. Schönhense, Magnetic circular dichroism in valence-band photo-emission from Fe(100). *J. Magn. Magn. Mater.* **104-107**, 1691–1692 (1992).
37. J. Stöhr, Y. Wu, B. D. Hermsmeier, M. G. Samant, G. R. Harp, S. Koranda, D. Dunham, B. P. Tonner, Element-specific magnetic microscopy with circularly polarized x-rays. *Science* **259**, 658–661 (1993).
38. G. van der Laan, B. T. Thole, Spin polarization and magnetic dichroism in photoemission from core and valence states in localized magnetic systems. II. Emission from open shells. *Phys. Rev. B* **48**, 210–223 (1993).
39. J. Braun, The theory of angle-resolved ultraviolet photoemission and its applications to ordered materials. *Rep. Prog. Phys.* **59**, 1267–1338 (1996).
40. H. Ebert, J. Schwitalla, Magnetic dichroism in valence-band x-ray photoemission spectroscopy. *Phys. Rev. B* **55**, 3100 (1997).
41. J. Henk, T. Scheunemann, S. V. Halilov, and R. Feder, Magnetic dichroism and electron spin

polarization in photoemission: Analytical results. *J. Phys. Condens. Matter* **8**, 47–65 (1996).

42. T. Yokoyama, T. Nakagawa, Y. Takagi, Magnetic circular dichroism for surface and thin film magnetism: Measurement techniques and surface chemical applications. *Int. Rev. Phys. Chem.* **27**, 449–505 (2008).
43. K. Hild, J. Maul, G. Schönhense, H. J. Elmers, M. Amft, P. M. Oppeneer, Magnetic circular dichroism in two-photon photoemission. *Phys. Rev. Lett.* **102**, 057207 (2009).
44. T. Berlijn, P. Snijders, O. Delaire, H.-D. Zhou, T. Maier, H.-B. Cao, S.-X. Chi, M. Matsuda, Y. Wang, M. Koehler, P. R. C. Kent, H. H. Weitering, Itinerant antiferromagnetism in RuO_2. *Phys. Rev. Lett.* **118**, 077201 (2017).
45. Z. Zhu, J. Strempfer, R. Rao, C. Occhialini, J. Pelliciari, Y. Choi, T. Kawaguchi, H. You, J. Mitchell, Y. Shao-Horn, R. Comin, Anomalous antiferromagnetism in metallic RuO_2 determined by resonant x-ray scattering. *Phys. Rev. Lett.* **122**, 017202 (2019).
46. K.-H. Ahn, A. Hariki, K.-W. Lee, and J. Kuneš, Antiferromagnetism in RuO_2 as d-wave Pomeranchuk instability. *Phys. Rev. B* **99**, 184432 (2019).
47. V. Jovic, R. J. Koch, S. K. Panda, H. Berger, P. Bugnon, A. Magrez, K. E. Smith, S. Biermann, C. Jozwiak, A. Bostwick, E. Rotenberg, S. Moser, Dirac nodal lines and flat-band surface state in the functional oxide RuO_2. *Phys. Rev. B* **98**, 241101 (2018).
48. K. Medjanik, O. Fedchenko, S. Chernov, D. Kutnyakhov, M. Ellguth, A. Oelsner, B. Schönhense, T. R. F. Peixoto, P. Lutz, C.-H. Min, F. Reinert, S. Däster, Y. Acremann, J. Viefhaus, W. Wurth, H. J. Elmers, G. Schönhense, Direct 3D mapping of the Fermi surface and Fermi velocity. *Nat. Mater.* **16**, 615–621 (2017).
49. C. Westphal, J. Bansmann, M. Getzlaff, G. Schönhense, Circular dichroism in the angular distribution of photoelectrons from oriented CO molecules. *Phys. Rev. Lett.* **63**, 151–154 (1989).
50. H. Daimon, T. Nakatani, S. Imada, S. Suga, Circular dichroism from non-chiral and non-magnetic materials observed with display-type spherical mirror analyzer. *J. Electron Spectrosc. Relat. Phenom.* **76**, 55–62 (1995).
51. H. Ebert, D. Ködderitzsch, J. Minár, Calculating condensed matter properties using the KKR-Green's function method—Recent developments and applications. *Rep. Prog. Phys.* **74**, 096501 (2011).
52. J. Braun, J. Minár, H. Ebert, Correlation, temperature and disorder: Recent developments in the one-step description of angle-resolved photoemission. *Phys. Rep.* **740** 1–34 (2018)
53. L. Šmejkal, A. Marmodoro, K.-H. Ahn, R. Gonzalez-Hernandez, I. Turek, S. Mankovsky, H. Ebert, S.

W. D'Souza, O. Šipr, J. Sinova, T. Jungwirth, Chiral magnons in altermagnetic RuO_2. arXiv:2211.13806 [cond-mat.mes-hall] (2022).

54. M. R. Scholz, J. Sánchez-Barriga, J. Braun, D. Marchenko, A. Varykhalov, M. Lindroos, Y. J. Wang, H. Lin, A. Bansil, J. Minár, H. Ebert, A. Volykhov, L. V. Yashina, O. Rader, Reversal of the circular dichroism in angle-resolved photoemission from $Bi_2 Te_3$. *Phys. Rev. Lett.* **110**, 216801 (2013), .
55. J. Baruchel, M. Schlenker, and B. Barbara, 180° Antiferromagnetic domains in MnF_2 by neutron topography. *J. Magn. Magn. Mater.* **15-18**, 1510–1512 (1980).
56. G. Schönhense, Circular dichroism and spin polarization in photoemission from adsorbates and non-magnetic solids. *Phys. Scr.* **1990**, 255–275 (1990).
57. G. Derondeau, F. Bisti, M. Kobayashi, J. Braun, H. Ebert, V. A. Rogalev, M. Shi, T. Schmitt, J. Ma, H. Ding, V. N. Strocov, J. Minár, Fermi surface and effective masses in photoemission response of the $(Ba_{1-x} K_x)Fe_2As_2$ superconductor. *Sci. Rep.* **7**, 8787 (2017).
58. O. Stejskal, M. Veis, J. Hamrle, Band structure analysis of the magneto-optical effect in bcc Fe. *Sci. Rep.* **11**, 21026 (2021).
59. H. Ebert, A. Perlov, S. Mankovsky, Incorporation of the rotationally invariant LDA + U scheme into the SPR-KKR formalism: Application to disordered alloys. *Solid State Commun.* **127**, 443–446 (2003).
60. P. Lloyd, Wave propagation through an assembly of spheres: II. The density of single-particle eigenstates. *Proc. Phys. Soc.* **90**, 207–216 (1967).
